# Significant Measures of Gaze and Pupil Movement for Evaluating Empathy between Viewers and Digital Content

**DOI:** 10.3390/s22051700

**Published:** 2022-02-22

**Authors:** Jing Zhang, Sung Park, Ayoung Cho, Mincheol Whang

**Affiliations:** 1 Department of Emotion Engineering, Sangmyung University, Seoul 03016, Korea; zj03010020@gmail.com (J.Z.); spica7601@gmail.com (S.P.); joa6391@gmail.com (A.C.); 2 Department of Human-Centered Artificial Intelligence, Sangmyung University, Seoul 03016, Korea

**Keywords:** empathy evaluation, eye movement, gaze, pupil, fixation, saccade, emotion, cognition, digital content

## Abstract

The success of digital content depends largely on whether viewers empathize with stories and narratives. Researchers have investigated the elements that may elicit empathy from viewers. Empathic response involves affective and cognitive processes and is expressed through multiple verbal and nonverbal modalities. Specifically, eye movements communicate emotions and intentions and may reflect an empathic status. This study explores feature changes in eye movements when a viewer empathizes with the video’s content. Seven feature variables of eye movements (change of pupil diameter, peak pupil dilation, very short, mid, over long fixation duration, saccadic amplitude, and saccadic count) were extracted from 47 participants who viewed eight videos (four empathic videos and four non-empathic videos) distributed in a two-dimensional emotion axis (arousal and valence). The results showed that viewers’ saccadic amplitude and peak pupil dilation in the eigenvalues of eye movements increased in the empathic condition. The fixation time and pupil size change showed limited significance, and whether there were asymmetric pupil responses between the left and right pupils remained inconclusive. Our investigation suggests that saccadic amplitude and peak pupil dilation are reliable measures for recognizing whether viewers empathize with content. The findings provide physiological evidence based on eye movements that both affective and cognitive processes accompany empathy during media consumption.

## 1. Introduction

We live in a society with an overflow of media content through various media forms. Digital content consists of a stream of information in digital format that can be stored, streamed, and broadcast. Whereas digital content may include data devoid of any affective characteristics (e.g., weather information and geological information), some content, such as drama and movies, highly depends on its emotional value.

Digital content has a spectrum of affective characteristics depending on the purpose of the medium (drama, movie, ads). Most digital content shares a common and permeating goal to produce media that many viewers can relate to, understand, and engage emotionally. For example, the Netflix program most viewed in 2021 was South Korea’s *Squid Game*. People argue that *Squid Game* became popular because viewers readily empathize with a character’s emotional state and narrative. The psychology and physiology of empathy have long been studied in the fields of clinical psychology, social development, and neuroscience. While there is no consensus on the definition of empathy, researchers agree that empathy has multiple subcomponents [1,2,3], and some critical elements of empathy (e.g., recognition, process, outcome, and response) are commonly identified (for an extensive review of empathy as a concept, see [4]).

Based on the most prominent empathy theories [1,3,5,6], affective and cognitive processes are the underlying mechanisms that produce empathic outcomes. Affective empathy generally connotes an observer’s visceral reaction to the target’s affective state. Cognitive empathy involves taking the target’s perspective and drawing inferences about their thoughts, feelings, and characteristics.

Neuroscientists have identified underlying neurological evidence for empathy [7] by discovering mirror neurons in monkeys [8]. Overlapping brain patterns are observed when an observer perceives the same emotions from a target, suggesting shared affective neural networks [9,10,11]. In this paper, we first discuss related work, summarizing significant gaze and pupil movement measures and comparing eye movement studies on digital content. We then explain our experiment design and protocol, followed by data analysis. We conclude with a discussion on the implications of the findings, the limitations of the study, and call for future research.

## 2. Related Work

Attention to visual information is a prerequisite for recognition. The cortical area known as the frontal eye field (FEF) plays a vital role in controlling visual attention and eye movements [12]. The fovea on the retina is only a relatively small part, but it contains sufficiently dense cone cells to distinguish the visual world in great detail [13]. Owing to the relatively small fovea, the brain makes significant decisions when controlling eye movements. A saccade is a decision each time we move our eyes, and we have to decide where and when to move them [14,15]. Personalities, desires, goals, beliefs, expectations, predictions, memories, and intentions can influence these decisions.

Gaze is a potent social cue in which mutual gaze often signifies threat or evading conveying submission or avoidance [16,17,18]. Processing eye gaze is a foundation for social interactions because explication of the neural substrate for gaze processing is an important step in understanding neuroscience for social cognition [19,20]. Gaze tracking monitors the user’s attention and interests and personalizes the agent’s behaviors [21], which is an essential tool for detecting users’ attention information and focusing on particular content. It is critical to analyze consumers’ attention when an advertisement is shown [22].

Researchers have long confirmed through empirical evidence that eyes can perceive and express emotions. A classic study by Hess [23] demonstrated that pleasant imagery leads to pupil dilation. The relationship between pupil modulation and emotion perception develops with age [24]. Pupil size is generally regarded as a nonverbal communication channel in which social signals are exchanged between individuals at an unconscious level (i.e., non-reportable). Specifically, a person’s feelings or attitudes are embedded in pupil size as a source of information [25]. Involuntary pupil size change is also regulated by the autonomic nervous system.

Pupil dilation seems to occur when people feel attracted [25], surprised or uncertain [26], or social interest. Active storage or retrieval of memories also leads to pupil dilation and an increase in cognitive load [27,28]. A pleasant emotion leads to pupil dilation more than an unpleasant one [24].

In the context of empathy, the dilation pattern seems to get synchronized between conversation partners if the dyadic pair shares attention (i.e., “tunes in”) and gets engaged, evident in the shared emotional peak found in a video analysis by Kang [29]. Kang also found that pupil synchronization was the strongest among the high-expressive and high-empathic participant groups. Pupil synchronization also interacts with the degree of trust [30] and facial expression of the conversation pair [31]. For example, sad faces elicit more pupil synchronization than happy faces.

In short, the analysis of eye movement features is critical for understanding the degree of empathy among individuals. Eye features (i.e., gaze and pupil movement) change when an observer empathizes with an individual. However, research on whether eye features change when empathizing with content is in its infancy. Table 1 compares the most recent studies on eye movement features when viewing media. There is little research analyzing eye movement features between a person and media suggesting key indicators for use. Furthermore, except for [32], no study has investigated the relationship between gaze and pupil movement for evaluating empathy. In addition, the dependent measures of most studies are limited to a single index (e.g., only they investigated gaze points or time spent of fixation).

Our study sought to identify significant gaze and pupil movement measures for assessing empathy between viewers and digital content. To the best of our knowledge, this is the first study to investigate the relationship between significant gaze and pupil movements and empathic digital content. Second, the study analyzes a full range of significant measures involving gaze and pupil movements (change of pupil diameter, peak pupil dilation, very short, mid, over long fixation duration, saccadic amplitude, and saccadic count) for use when assessing digital content.

## 3. Materials and Methods

We adopted Russell’s two-dimensional model [37], where emotional states can be defined at any valence and arousal level. We invited participants to view empathic or non-empathic emotion-eliciting videos with varying valence (i.e., from unpleasant to pleasant) and arousal levels (i.e., from relaxed to aroused). Our research aimed to verify the following nine hypotheses. Based on the aforementioned literature review, we hypothesized a significant difference in eye movement features (pupil size, fixation, and saccade) when a person views digital content:

**Hypothesis** **1** **(H1).** *… between the empathic and non-empathic conditions in all videos (i.e., pleasant-aroused, pleasant-relaxed, unpleasant-aroused, and unpleasant-relaxed)*.

**Hypothesis** **2** **(H2).** *… between empathic and non-empathic conditions in aroused videos*.

**Hypothesis** **3** **(H3).** *… between empathic and non-empathic conditions in relaxed videos*.

**Hypothesis** **4** **(H4).** *… between empathic and non-empathic conditions in pleasant videos*.

**Hypothesis** **5** **(H5).** *… between empathic and non-empathic conditions in unpleasant videos*.

**Hypothesis** **6** **(H6).** *… between empathic and non-empathic conditions in pleasant-aroused videos*.

**Hypothesis** **7** **(H7).** *… between empathic and non-empathic conditions in pleasant-relaxed videos*.

**Hypothesis** **8** **(H8).** *… between empathic and non-empathic conditions in unpleasant-relaxed videos*.

**Hypothesis** **9** **(H9).** *… between empathic and non-empathic conditions in unpleasant-aroused videos*.

### 3.1. Stimuli Selection

In this study, we edited video clips (e.g., dramas or movies) to elicit empathy from the participants. The content to induce empathic conditions was collected in a two-dimensional model. To ensure that the empathic and non-empathic videos were effective, we conducted a stimulus selection experiment before the main experiment. We selected 20 edited dramas or movies containing emotions as candidates. Five video clips were used for each quadrant in a two-dimensional model. Thirty participants viewed emotional videos and responded to a subjective questionnaire. They received $20 for participation in the study. For each condition, among the five candidates, the video with the highest empathic score was selected as the empathic stimulus in the main experiment. Conversely, the video with the lowest empathic score was chosen as the non-empathic stimulus. That is, a pair of empathic and non-empathic videos for each of the four quadrants in the two-dimensional model was selected. In total, eight stimuli were selected for the main experiment. All stimuli are available online (see Supplementary Materials).

### 3.2. Experiment Design

When the observer is interested in the target stimulus, the observer’s eye movement characteristics change as a function of the target’s emotional characteristics (empathy, valence, and arousal). To understand the nature of such a change, the main experiment was a factorial design of two (empathy: empathic and non-empathic) × two (valence: pleasant and unpleasant) × two (arousal: aroused and relaxed) independent variables. A *t*-test was used to test the difference in eye movement-related dependent measures (pupil size, fixation, and saccade) between the empathic and non-empathic conditions.

### 3.3. Participants

We conducted an a priori power analysis using the program G*Power with power set at 0.8 and α = 0.05, d = 0.6 (independent *t*-test), two-tailed. The results suggest that an N of approximately 46 is needed to achieve appropriate statistical power. Therefore, 47 university students were recruited for this study. Participants’ ages ranged from 20 to 30 years (mean = 28, STD = 2.9), with 20 (44%) men and 27 (56%) women. We selected participants with a corrective vision of 0.8 or above without any vision deficiency, to ensure reliable recognition of visual stimuli. We recommended that participants sleep sufficiently and prohibited alcohol, caffeine, and smoking the day before the experiment. Because the experiment requires valid recognition of the participant’s facial expression, we limited the use of glasses and cosmetic makeup. All participants were briefed on the purpose and procedure of the experiment and signed a consent form. They were then compensated for their participation with a fee paid to them.

### 3.4. Experimental Protocol

**Figure 1:**
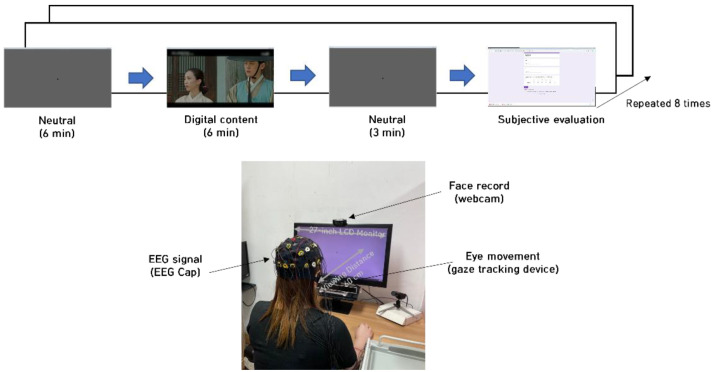
Experimental protocol and configuration.

Figure 1 outlines the experimental process and the environment used in this study. Participants were asked to sit 1 m away from a 27-inch LCD monitor. A webcam was installed on the monitor. Participants’ brainwaves (EEG cap 18 ch), facial expressions (webcam), and eye movements (gaze tracking device) were acquired in addition to subjective responses to a questionnaire. We set the frame rate of the gaze tracking device to 60 frames per second. The participants viewed eight emotion-eliciting (empathy or non-empathy) videos and responded to a questionnaire after each viewing. We excluded the brainwave data from the analysis in this paper.

We gathered the participants’ subjective responses using the Consumer Empathic Response to Advertising Scale (CERA), a comprehensive battery of measures involving affective and cognitive facets of empathy [38,39,40]. We adopted an empirically validated questionnaire based on the ethnicity of Korean participants, which consisted of nine items (see Table 2). The factor loading exceeded 0.4, and the Cronbach’s alpha exceeded 0.8. Each construct was measured on a seven-point Likert scale.

### 3.5. Feature Extraction of Eye Movement

Eye movement features play a vital role in face processing and social communication [41,42]. It is one of the most important facial cues for communicating with consumers [43,44]. Eye gaze direction is associated with viewer cognition, such as visual attention and emotion. Gaze movements convey emotions and intentions and can reflect empathic conditions. We selected seven feature measures of gaze movement and pupil characteristics for the extraction and analysis, as outlined in Table 3. We did not measure pupil response time and decision time.

#### 3.5.1. Change in Pupil Diameter

Pupillometry, the measurement of changes in pupil diameter, is a relatively old method for inferring different types of activity in the brain. Pupil dilation is an autonomic sympathetic nervous system response that can provide attention, interest, or emotion indices, and is correlated with mental workload and arousal [45].

Pupil responses may be a useful alternative or an addition to subjective measures. Some cognitive and emotional events occur outside our conscious control and can cause pupils to constrict and expand. UX researchers have recorded data from these events to detect fear, anxiety, mental strain, or task difficulty [46]. In addition, because it is nearly impossible to mask implicit cognitive responses, biases such as social desirability that prevent people from accurately informing researchers of their experiences are of little concern during analysis.

Chatham, Frank, and Munakata [47] established the utility of both pupillometry for assessing the temporal dynamics of cognitive control. Changes in central nervous system activity that are systematically related to cognitive processing may be extracted from the raw pupillary record by performing time-locked averaging of critical events in the information-processing task. A task-evoked pupillary response bears the same relationship to the pupillary record from which it is derived, as does an event-related brain potential to spontaneous electroencephalographic (EEG) activity. With averaging, short-latency (i.e., from onset between 100 and 200 ms) phasic task-evoked dilations appear, which terminate rapidly following the completion of processing [48]. In pupillometry, participants were calibrated and then looked at a fixation cross on a blank page for one second to obtain a baseline pupil diameter measurement [49].

We were interested in the relationship between pupil size and the empathic and non-empathic video conditions. Since there is evidence that the left and right pupils may be different [50], we explored the possible differences in the responses of the left and right pupils. The perception-action model is adopted by many fields over time, and perception and action share a common code of representation in the brain [51,52]. The left hemisphere processes detailed information, whereas the right hemisphere is selective for more holistic information [53]. The left prefrontal area is more active in response to semantic cues, whereas the right prefrontal area is more active in generating information from memory. Both are active when the task requires voluntary or imagined actions [54,55]. While the left hemisphere subserves positive emotions, the right hemisphere may subserve fearful or negative emotions [56,57]. Owing to such differential activation as a function of emotion and because pupil sizes reflect brain activity, we speculate that pupil diameter changes may differ between empathic and non-empathic conditions. We calculated the mean baseline pupil diameter for each participant. Specifically, the change in the left pupil diameter (CLPD) and change in the right pupil diameter (CRPD) before and during the stimulus. We calculated the mean values of the CLPD and CRPD across all participant data as dependent measures.

#### 3.5.2. Peak of Pupil Dilation

The decision-making process drives the time course of pupil response. The pupil response reveals the properties of the decisions, such as perceived emotional valence and confidence in the assessment [47,58]. Beatty [48] reviewed all empirical data involving task-evoked pupillary response (TEPR) studies. He concluded that it took six to eight seconds for the participants to recognize and respond during cognitive tasks. The most prominent TEPR research [59,60] has set the pupil dilation experiment’s window size to eight seconds. We also set the window size to be eight seconds because empathic response involves a cognitive process [5]. The phase for extracting the peak value of pupil dilation was divided into three steps.

Step one: identifying the peak every eight seconds

**Figure 2:**
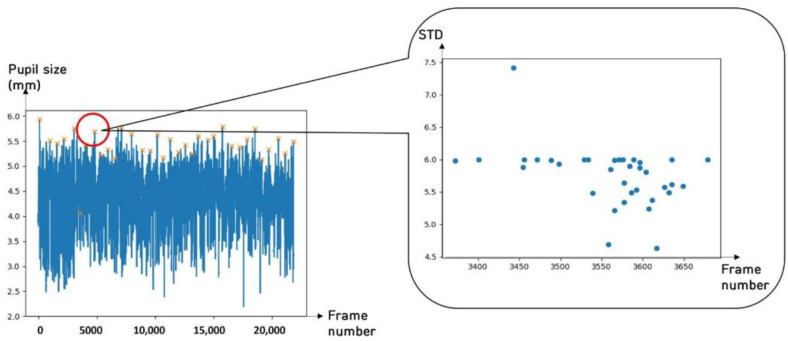
Identifying the peak every eight seconds in a video.

Figure 2 shows a schematic diagram of the peaks found every eight seconds in the raw data. However, peaks every eight seconds may contain false peaks. To counter false peaks, we compared the standard deviation (STD) of the peak positions of all 47 participants.

Step two: find the true peak

**Figure 3:**
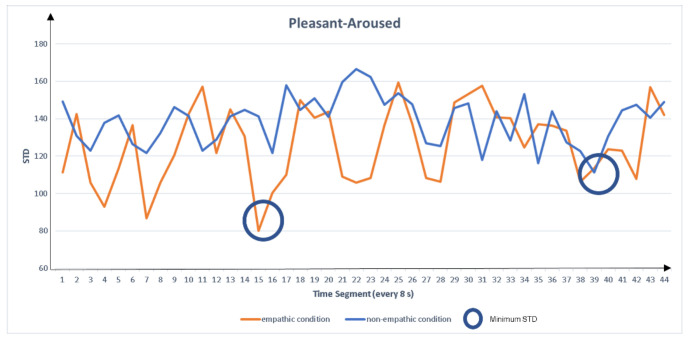
Comparison of standard deviation (STD) of peak pupil dilation between the empathic and non-empathic conditions in pleasant-aroused content.

**Figure 4:**
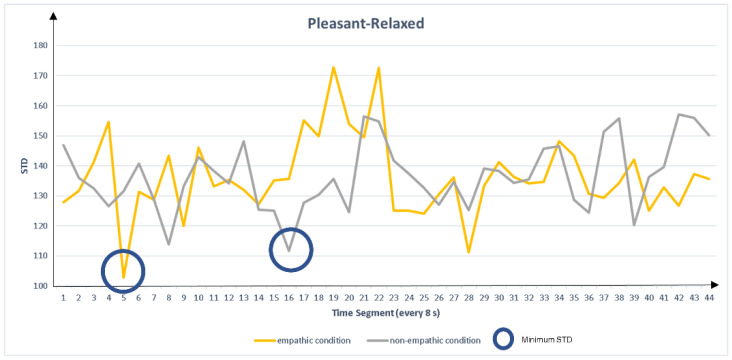
Comparison of standard deviation (STD) of peak pupil dilation between the empathic and non-empathic conditions in pleasant-relaxed content.

**Figure 5:**
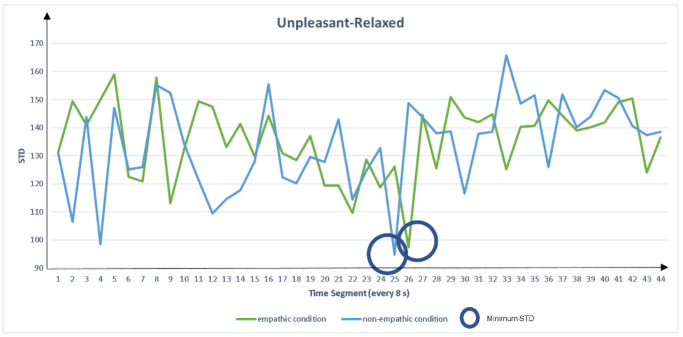
Comparison of standard deviation (STD) of peak pupil dilation between the empathic and non-empathic conditions in unpleasant-relaxed content.

**Figure 6:**
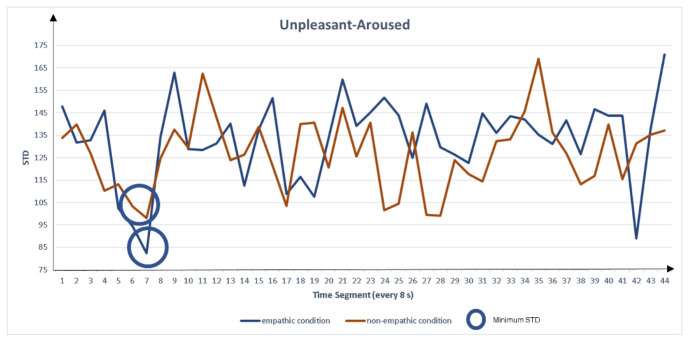
Comparison of standard deviation (STD) of peak pupil dilation between the empathic and non-empathic conditions in unpleasant-aroused content.

Because the peak with the smallest dispersion has the highest probability of being a true peak, we extracted the peak feature measures with the lowest STD for each empathic and non-empathic condition. The extracted measures were peak left pupil dilation (PLPD) and peak right pupil dilation (PRPD), as shown in Figure 3, Figure 4, Figure 5 and Figure 6. For the eigenvalue, we hypothesized that the maximum pupil dilation is greater in the empathic condition than in the non-empathic condition.

#### 3.5.3. Fixation Duration

The time between the two saccades is generally called fixation duration. This event is closely related to cognitive processing in alert subjects [61,62,63]. Fixations of different lengths may reflect different neuronal processes, as observed in various studies [64,65,66,67]. Very short fixations (<150 ms), so-called express fixations, may turn out to be a distinct category caused by low-level visuomotor behavior; they could represent the reflexive unconscious or noncognitive aspects of behavioral control.

Media-related fixation involves cognitive saccades (between 150 and 900 ms), positioned between very short (<150 ms) and overlong (>900 ms) saccades [68]. Medium fixation has a reduced fatigue rate compared to short or long fixation [69,70,71]. Galley and Andres [71] reported that visual processing of complex scenes with rapidly changing stimuli (e.g., city rides) typically leads to a fixation of between 200–400 ms, which exceeds the fixation duration of approximately 250 ms during reading. Fixation is associated with content-related identification or cognitive processing; therefore, we focused on fixation duration, ranging from 150 ms to 900 ms, in this study. A short fixation time (150 ms) is insufficient to extract relevant information [65]. In the case of excessively long fixation (>900 ms), a general functional interpretation has not yet been established, except for unconscious driving or low-arousal phase starting during microsleep.

Three eigenvalues were extracted: very short fixation, medium fixation duration, and overlong fixation. We calculated the percent dependent measure (%), which represents the fixed time divided by total time. We speculated that empathic videos may elicit more cognitive engagement and increase medium fixation than the non-empathic videos. 

#### 3.5.4. Saccade

Experimental studies of saccadic eye movements have produced a considerable amount of data. In the case of eye movements elicited by specific visual targets, the significant measures were the metrics of saccadic amplitude and saccadic count. The amplitude is the angle in degrees between two fixation points [61]. Measures were provided based on the calculation of GazePoint equipment, which averages eye positions. We hypothesized that the saccadic amplitude would be greater in the empathic condition than in the non-empathic condition.

## 4. Results

The results are twofold: the analysis of subjective evaluation and eye movement features.

### 4.1. Subjective Evaluation

A *t*-test was used to test the differences between the key features in the empathic and non-empathic conditions.

#### 4.1.1. The Analysis of Arousal Scores

**Figure 7:**
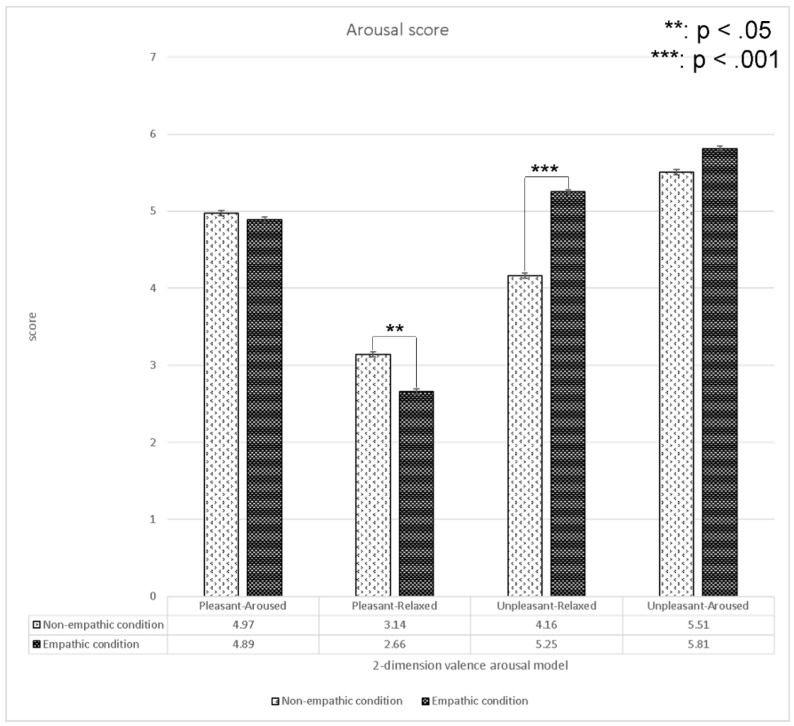
Analysis (*t*-test) of the arousal values between the empathic and non-empathic conditions.

We analyzed the differences in subjective arousal scores between the empathic and non-empathic conditions in four quadrants in the two-dimensional emotion model (i.e., pleasant-aroused, pleasant-relaxed, unpleasant-relaxed, and unpleasant-aroused; see Figure 7).

The results indicated that the arousal scores of the empathic condition in the pleasant-relaxed content were significantly lower than those in the non-empathic condition. Conversely, the arousal scores of the empathic condition in the unpleasant-relaxed content were significantly higher than those in the non-empathic condition. We found no significant difference between pleasant-aroused and unpleasant-aroused content.

#### 4.1.2. The Analysis of Valence Scores

**Figure 8:**
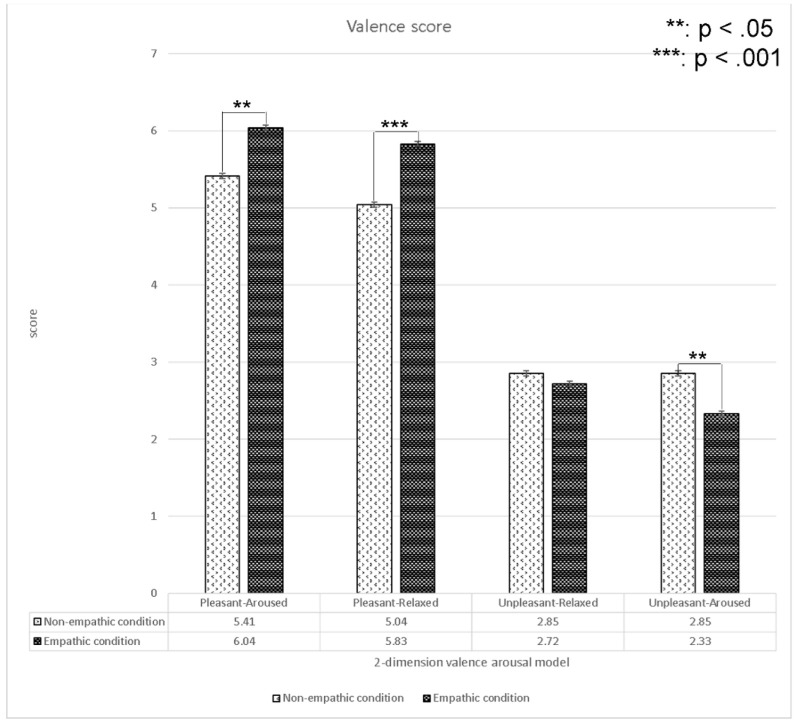
Analysis (*t*-test) of the valence values between the empathic and non-empathic conditions.

We analyzed the differences in subjective valence scores between the empathic and non-empathic conditions (see Figure 8). The results indicated that the valence scores of the empathic condition in the pleasant-aroused and pleasant-relaxed content were significantly higher than those in the non-empathic condition. Conversely, the valence scores of the empathic condition in the unpleasant-aroused content were significantly lower than those of the non-empathic condition. We found no significant differences in the unpleasant-relaxed content.

#### 4.1.3. The Analysis of Cognitive and Affective Empathy Scores

**Figure 9:**
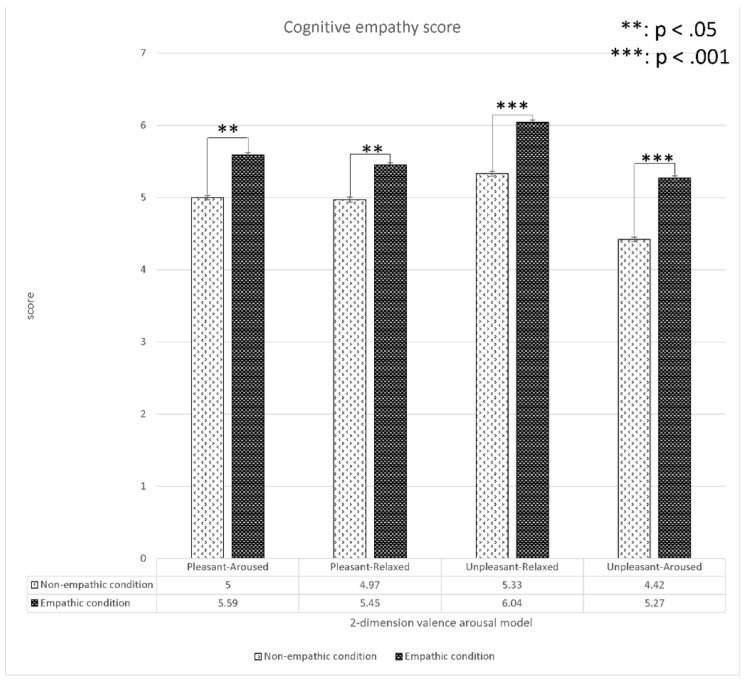
Analysis (*t*-test) of the cognitive empathy values between the empathic and non-empathic conditions.

**Figure 10:**
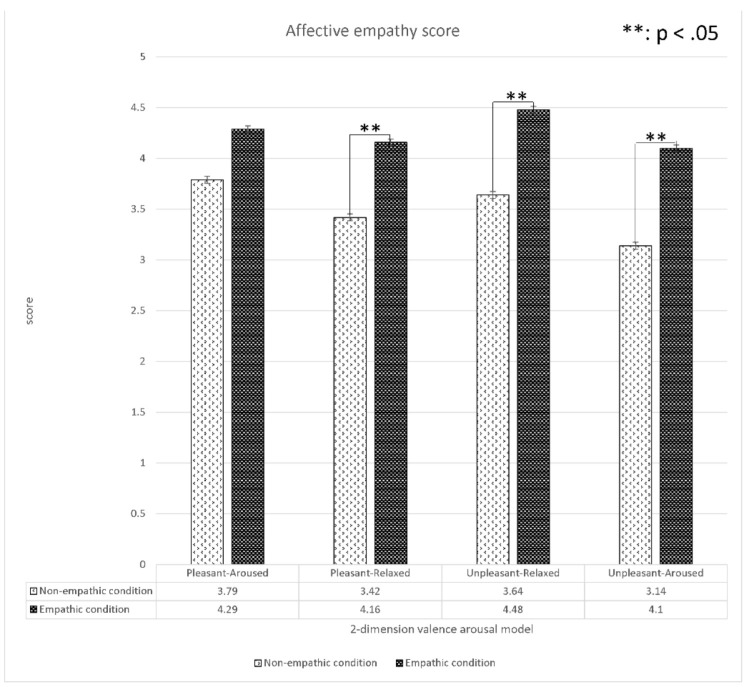
Analysis (*t*-test) of the affective empathy values between the empathic and non-empathic conditions.

We analyzed the differences in subjective cognitive and affective scores between the empathic and non-empathic conditions (Figure 9 and Figure 10). The results indicated that the cognitive empathy scores of the empathic condition in all four contents were significantly higher than those of the non-empathic condition. Similarly, the affective empathy scores of the empathic condition in all content except for pleasant-aroused content were significantly higher than those of the non-empathic condition. In summary, all empathic videos induced target empathy (empathic or non-empathy) in general from the participants as intended.

### 4.2. Eye Movement Features

A *t*-test analysis of the hypotheses was conducted by adjusting alpha levels of 0.05 per test. The results of key features of the nine groups are listed in Table 4, Table 5, Table 6 and Table 7. Overall, the saccadic amplitude measure (i.e., the mean angle between two fixation points) showed that, except for aroused and relaxed content, it is significantly greater in the empathic condition than in the non-empathic condition. In addition, pupil dilation showed a significant increase in the empathic condition compared to the non-empathic condition with aroused and pleasant content. The peak of pupil dilation ranged from 5.70 mm to 5.88 mm in the empathic condition. The detailed analysis of each content group follows.

#### 4.2.1. All-Emotions Content

For all-emotions content, results indicated the peak of right pupil dilation (Table 5) and saccadic amplitude (Table 7) were significantly different between the empathic and non-empathic conditions (*p* < 0.001). Interestingly, the saccadic amplitude was greater in the empathic condition (M = 193.74, STD = 2.45; *p* < 0.001) than in the non-empathic condition (M = 165.86, STD = 3.12; *p* < 0.001; see Table 7). However, the saccadic count did not show a significant difference. The fixation time in all three ranges (very short, medium, overlong) did not show a significant difference either.

The results indicated that the higher the level of empathic condition, the more active the saccadic jump, which may imply that empathic content is more interesting and engaging than non-empathetic content, i.e., viewers engage more in cognitive and attentive processes.

#### 4.2.2. Pleasant and Unpleasant Content

For pleasant content, the results indicated that the peak of left and right pupil dilation, and saccadic amplitude were significantly different between the empathic and non-empathic conditions (*p* < 0.05). This is consistent with the literature on pupil dilation due to pleasant images [25] and happy facial expressions [24].

For unpleasant content, the mean of medium fixation was significantly smaller in the non-empathic condition (M = 610.11, STD = 12.48; *p* < 0.05) than in the empathic content (M = 649.50, STD = 12.36; *p* < 0.05). In addition, the mean of overlong fixation was smaller in the empathic condition (M = 51.38, STD = 2.25; *p* < 0.05) than in the non-empathic condition (M = 59.58, STD = 3.0; *p* < 0.05).

#### 4.2.3. Aroused and Relaxed Content

For aroused content, results indicated that the change in left and right pupil diameter, and peak left and right pupil dilation were significantly different between the empathic and non-empathic conditions (*p* < 0.05). Specifically, the change in left pupil diameter was significantly higher in the non-empathic condition (M = 0.39, STD = 0.06; *p* < 0.01) than that in the empathic condition (M = 0.22, STD = 0.04; *p* < 0.01). In addition, the change in right pupil diameter was significantly higher in the non-empathic condition (M = 0.38, STD = 0.06; *p* < 0.05) than in the empathic condition (M = 0.29, STD = 0.05; *p* < 0. 05). Overall, the significant pupil dilation is limited to pleasant and aroused conditions. The implications will be discussed in Discussion.

For relaxed content, results indicated that the change in left pupil diameter, overlong fixation, and saccadic amplitude were significantly different between the empathic and non-empathic conditions.

#### 4.2.4. Empathic and Non-Empathic Content

**Figure 11:**
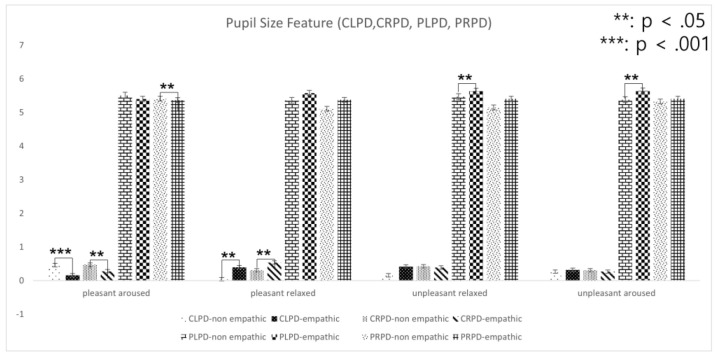
Pupil feature variables in a two-dimensional emotion map.

**Figure 12:**
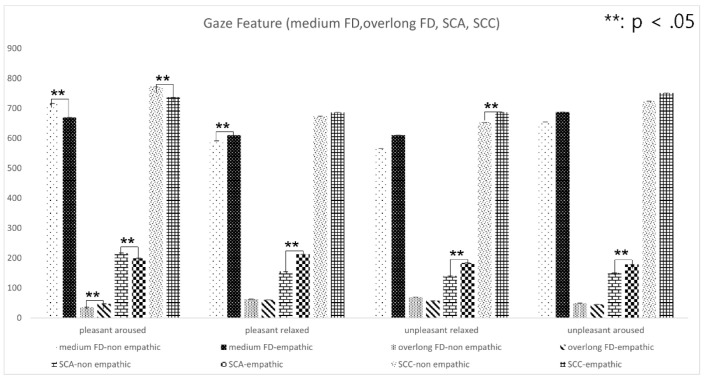
Gaze feature variables in a two-dimensional emotion map.

Figure 11 and Figure 12 depict the relationship between eye-movement feature variables in a two-dimensional emotion map. For pleasant-aroused content, the results indicated that changes in left and right pupil diameter, peak left pupil dilation, medium fixation duration, overlong fixation duration, saccadic amplitude, and saccadic count were significantly different between the empathic and non-empathic conditions (*p* < 0.05).

For pleasant-relaxed content, the results indicated that the changes in left and right pupil diameter, medium fixation duration, and saccadic amplitude were significantly different between the empathic and non-empathic conditions (*p* < 0.05).

For unpleasant-relaxed content, the results indicated that peak left pupil dilation, medium fixation duration, saccadic amplitude, and saccadic count were significantly different between the empathic and non-empathic conditions (*p* < 0.05).

For unpleasant-aroused content, the results indicated that PLPD and saccadic amplitude were significantly different between the empathic and non-empathic conditions (*p* < 0.05).

## 5. Conclusions and Discussion

To the best of our knowledge, this is the first study to suggest significant measures involving gaze and pupil movements for use when assessing empathic digital content. We analyzed a full range of dependent measures (change in pupil diameter, peak pupil dilation, very short, mid, overlong fixation duration, saccadic amplitude, and saccadic count) to understand all aspects of gaze and pupil movements. Our study had more indices than other studies (Table 1).

The majority (H_1_, H_3_, H_4_, H_5_, H_6_) of the hypotheses on peak pupil dilation and saccadic amplitude were supported. In conclusion, we found that saccadic amplitude and peak pupil dilation are two significant measures that can be used to assess whether viewers empathize with digital content.

Saccadic amplitude measures showed that, except for aroused and relaxed content, the average angle between two fixation points is significantly greater in the empathic condition than in the non-empathic condition. Because the empathic video was designed to induce empathy, as confirmed by the manipulation check, participants may have engaged in the story or narrative of the stimuli video (e.g., drama or movie). Participants may be “tuned” into digital content and initiate active information-seeking behavior, which results in a more dynamic saccadic jump in the region of interest. Participants also had a longer fixation with the empathic video than with non-empathic videos, albeit only with unpleasant and pleasant videos.

Second, although not as substantial as with saccadic amplitude, pupil dilation showed a significant increment in the empathic condition compared to the non-empathic condition with aroused and pleasant videos. In general, pupil dilation increases when the pupil is attracted or interested [25]. Empathic videos may certainly have drawn more attention. However, it is paramount to note that a higher form of empathy includes perspective taking [72]. Some stimuli may have induced the participants to refer to their past memories to understand the narrative. Memory retrieval is known to elicit pupil dilation [73], leading to cognitive load [27].

It is also interesting that differential pupil dilation between empathic and non-empathic conditions is limited to aroused and pleasant videos. This may be because of the main effect of pleasant images [25] and happy facial expressions [24] on pupil dilation. That is, other videos (unpleasant, relaxed) may have offset the dilation owing to the nature of the video. Further studies may design a more sensitive experiment with substantial statistical power.

Third, we did not find conclusive evidence suggesting asymmetric pupil responses when viewing empathic digital content. This is consistent with the current literature suggesting pupil-size asymmetry as a physiological trait (e.g., gender, personality) [74] or limited to cases such as migraine and headache [75].

We acknowledge some limitations of the research. First, we have yet to unravel the physiological mechanisms behind the findings. Future studies may investigate the relationship between brainwaves and gaze movement through EEG data analysis.

Second, the videos were not qualitatively analyzed (for example, the identification of emotional peaks, analysis of the actor’s facial expressions) to cross-examine the content and the participants’ responses. Empathy is a co-social behavior between a dyadic pair; looking into the relationship between the content and the change in the participant’s gaze and eye movements in a time series merits further investigation.

Third, the current study acquired the gaze data through an eye tracking device, but future research may obtain data through a camera for better usability and ecological validity. For example, Naqvi et al. [76] proposed a fuzzy system-based target selection method for target selection for camera-based gaze trackers. The results suggested better usability and performance than other gaze tracking methods. The fuzzy system uses three features (pupil size, gaze position, texture information of a monitor image at the gaze target) to decide the user’s target selection. Future studies to understand the participant’s empathic gaze movement may adopt such state-of-the-art camera-based gaze tracking methods.

## Figures and Tables

**Table 1:** Comparison of previous and proposed methods.

Methods	Strengths	Weaknesses
The physical elements of the advertisements (e.g., color, saturation, and value) and the viewer’s region of interest (ROI) were analyzed through gaze tracking [32].	The physical characteristics of the advertisements that elicit empathy were investigated.	The study focused on the media’s physical characteristics, not necessarily on the viewer.
A gaze-points prediction method for advertising images was proposed. The method includes a CNNs-based model for saliency prediction of the multi-text advertising images [33].	Analyzed the viewer’s attention based on the continuous distribution of gaze points when an ad is provided as a stimulus. The model adopted text enhanced learning to detect the multi-text peculiarity of ads.	The analysis is limited to a single index (e.g., gaze points) on attention to the advertisement.
Studied the overshadowing effect of a celebrity based on the analysis of advertisement effect based on fixation [34].	Analyzed the viewer’s fixation measures (time and count) on the celebrity and brand in an advertisement	The analysis is limited to a single index (e.g., time spent on fixation and fixation count) on attention to the advertisement
Analyzed the relationship between the quality of narrative rhetoric and the participant’s attention, duration, and pupil diameter [35].	Investigated print advertisements that used narrative techniques to present product effects (e.g., dramatic conflict).	The analysis is limited to a few indexes (e.g., gaze time and pupil diameter).
Analyzed differential visual attention to Facebook advertisements [36].	Investigated visual attention to ads viewed by different interpersonal relationships.	The analysis is limited to the fixation position, duration, and pupil magnification.
Empathy evaluation of gaze and pupil movement (**proposed method**).	In-depth analysis with all aspects of significant measures of gaze and pupil movement, including various frequencies involving fixation.	The understanding of the neurological mechanism is still absent. The stimulus’s subcomponents (e.g., celebrity, text) were not analyzed.

**Table 2:** Questionnaire of empathy, valence, and arousal.

NO.	Questionnaire	Factor
1	I felt pleasant as opposed to unpleasant	Valence
2	I felt aroused as opposed to relaxed	Arousal
3	I understood the characters’ needs	Cognitive empathy
4	I understood how the characters were feeling	
5	I understood the situation of the video	
6	I understood the motives behind the characters’ behavior	
7	I felt as if the events in the video were happening to me	Affective empathy
8	I felt as if I was in the middle of the situation	
9	I felt as if I was one of the characters	

**Table 3:** Gaze movement and pupil for extraction features.

Pupil Size Feature	Fixation Feature	Saccade Feature
Change in pupil diameter Peak pupil dilation	Very short fixation duration	Saccadic amplitude Saccadic count
	Mid-fixation duration	
	Over long fixation duration	

**Table 4:** The *t*-test analysis of the change in pupil diameter between the empathic and non-empathic conditions.

Group	Change in Left Pupil Diameter (CLPD)					Change in Right Pupil Diameter (CRPD)				
		Non-Empathic		Empathic			Non-Empathic		Empathic	
	*p*-Value	Mean	STD	Mean	STD	*p*-Value	Mean	STD	Mean	STD
All emotions	*p* > 0.1	0.31	0.04	0.24	0.06	*p* > 0.1	0.38	0.03	0.38	0.03
Aroused	*p* = 0.002	0.39	0.06	0.22	0.04	*p* = 0.026	0.38	0.06	0.29	0.05
Relaxed	*p* = 0.007	0.10	0.11	0.41	0.06	*p* > 0.1	0.37	0.04	0.46	0.05
Pleasant	*p* > 0.1	0.25	0.08	0.28	0.06	*p* > 0.1	0.40	0.04	0.41	0.05
Unpleasant	*p* > 0.1	0.21	0.08	0.37	0.06	*p* > 0.1	0.37	0.04	0.33	0.06
Pleasant aroused	*p* = 0.000	0.46	0.05	0.16	0.06	*p* = 0.008	0.48	0.05	0.28	0.08
Pleasant relaxed	*p* = 0.012	0.04	0.16	0.40	0.11	*p* = 0.005	0.31	0.06	0.54	0.06
Unpleasant relaxed	*p* > 0.1	0.16	0.15	0.42	0.06	*p* > 0.1	0.43	0.06	0.39	0.07
Unpleasant aroused	*p* > 0.1	0.27	0.06	0.32	0.11	*p* > 0.1	0.31	0.05	0.27	0.11

**Table 5:** The *t*-test analysis of the peak pupil dilation between the empathic and non-empathic conditions.

Group	Peak Left Pupil Dilation (PLPD)					Peak Right Pupil Dilation (PRPD)				
		Non-Empathic		Empathic			Non-Empathic		Empathic	
	*p*-Value	Mean	STD	Mean	STD	*p*-Value	Mean	STD	Mean	STD
All emotions	*p* > 0.1	5.41	0.03	5.46	0.04	*p* < 0.001	5.30	0.03	5.70	0.03
Aroused	*p* = 0.000	5.65	0.03	5.18	0.04	*p* = 0.000	5.21	0.04	5.78	0.02
Relaxed	*p* > 0.1	5.37	0.03	5.44	0.04	*p* = 0.000	5.21	0.06	5.88	0.02
Pleasant	*p* = 0.010	5.23	0.05	5.42	0.05	*p* = 0.000	5.41	0.03	5.73	0.05
Unpleasant	*p* > 0.1	5.57	0.03	5.49	0.05	*p* = 0.000	5.12	0.06	5.67	0.03
Pleasant aroused	*p* > 0.1	5.51	0.09	5.40	0.08	*p* = 0.045	5.52	0.07	5.27	0.09
Pleasant relaxed	*p* > 0.1	5.36	0.09	5.58	0.12	*p* > 0.1	5.11	0.10	5.38	0.10
Unpleasant relaxed	*p* = 0.012	5.47	0.17	5.64	0.07	*p* > 0.1	5.15	0.09	5.41	0.08
Unpleasant aroused	*p* = 0.007	5.38	0.07	5.65	0.08	*p* > 0.1	5.33	0.08	5.41	0.07

**Table 6:** The *t*-test analysis of the fixation duration between the empathic and non-empathic conditions.

Group	Very Short Fixation (<150 ms)					Medium Fixation (150 ms–900 ms)					Overlong Fixation (>900 ms)				
		Non-Empathic		Empathic			Non-Empathic		Empathic			Non-Empathic		Empathic	
	*p*-Value	Mean	STD	Mean	STD	*p*-Value	Mean	STD	Mean	STD	*p*-Value	Mean	STD	Mean	STD
All emotions	*p* > 0.1	18.02	0.71	17.26	0.66	*p* > 0.1	632.1	9.61	644.41	8.52	*p* > 0.1	54.69	2.12	52.82	1.61
Aroused	*p* > 0.1	18.59	1.09	17.82	0.98	*p* > 0.1	685.75	11.54	678.49	11.02	*p* > 0.1	42.90	2.43	46.68	2.20
Relaxed	*p* > 0.1	17.45	0.90	16.70	0.90	*p* > 0.1	579.0	13.26	611.3	12.02	*p* = 0.045	66.35	3.02	58.76	2.20
Pleasant	*p* > 0.1	18.20	1.12	16.90	0.93	*p* > 0.1	654.3	14.29	639.2	11.71	*p* > 0.1	49.74	2.91	54.26	2.31
Unpleasant	*p* > 0.1	17.84	0.87	17.61	0.95	*p* = 0.027	610.11	12.48	649.50	12.36	*p* = 0.031	59.58	3.00	51.38	2.25
Pleasant aroused	*p* > 0.1	18.51	1.74	18.69	1.48	*p* = 0.012	716.3	16.79	669.8	14.65	*p* = 0.001	36.44	3.07	47.71	3.00
Pleasant relaxed	*p* > 0.1	17.89	1.40	15.10	1.06	*p* = 0.08	592.29	19.28	609.95	17.10	*p* > 0.1	63.04	4.12	60.54	3.24
Unpleasant relaxed	*p* > 0.1	17.02	1.13	18.19	1.43	*p* > 0.1	566.0	18.03	610.29	17.07	*p* > 0.1	69.60	4.38	57.53	2.96
Unpleasant aroused	*p* > 0.1	18.68	1.33	17.04	1.26	*p* > 0.1	655.17	14.54	687.89	16.03	*p* > 0.1	49.36	3.53	45.37	3.16

**Table 7:** The *t*-test analysis on the saccadic amplitude and count between the empathic and non-empathic conditions.

Group	Saccadic Amplitude (SAC)					Saccadic Count (SCC)				
		Non-Empathic		Empathic			Non-Empathic		Empathic	
	*p*-Value	Mean	STD	Mean	STD	*p*-Value	Mean	STD	Mean	STD
All emotions	*p* < 0.001	165.86	3.12	193.74	2.45	*p* > 0.1	705.81	7.82	715.44	7.27
Aroused	*p* > 0.1	183.97	4.45	189.47	2.78	*p* > 0.1	748.25	9.41	744.01	9.19
Relaxed	*p* < 0.001	147.9	3.52	197.8	3.96	*p* > 0.1	663.82	10.86	687.78	10.47
Pleasant	*p* = 0.001	186.29	4.63	206.30	3.18	*p* > 0.1	723.2	11.98	711.3	9.87
Unpleasant	*p* = 0.000	145.65	2.98	181.32	3.27	*p* = 0.037	688.54	9.76	719.5	10.6
Pleasant aroused	*p* = 0.003	217.24	4.28	199.31	3.71	*p* = 0.019	772.2	14.34	737.2	12.19
Pleasant relaxed	*p* = 0.000	155.34	5.19	213.00	4.92	*p* > 0.1	674.23	16.31	686.52	14.54
Unpleasant relaxed	*p* = 0.000	140.7	4.54	183.54	5.45	*p* = 0.04	653.6	14.22	687.0	15.26
Unpleasant aroused	*p* = 0.000	150.70	3.73	179.13	3.63	*p* > 0.1	724.2	11.16	751.3	13.40

## Supplementary Materials

The following are available online at https://pan.baidu.com/s/1DmsCUAStDvKk_BHHnlwNrg?pwd=d8b2 (accessed on 31 December 2021). All videos used, and all raw data collected in the experiment.
